# A bibliometric analysis of immunotherapy for chronic hepatitis B: trends and hotspots prediction

**DOI:** 10.3389/fimmu.2026.1775211

**Published:** 2026-06-03

**Authors:** Xingtao Jin, Yiqiang Tang, Yanlu Ma, Man Li, Yueqiu Gao

**Affiliations:** 1 Institute of Cellular Immunity Laboratory, Shuguang Hospital Affiliated to Shanghai University of Traditional Chinese Medicine, Shanghai, China; 2 Institute of Shanghai Key Laboratory of Traditional Chinese Clinical Medicine, Shanghai, China; 3 Institute of Infectious Diseases, Shuguang Hospital Affiliated to Shanghai University of Traditional Chinese Medicine, Shanghai, China

**Keywords:** bibliometrics, chronic hepatitis B, functional cure, immune reconstitution, research trends

## Abstract

**Background:**

Chronic hepatitis B virus (HBV) infection represents a major global public health issue, affecting hundreds of millions worldwide and serving as a primary cause of cirrhosis and hepatocellular carcinoma. In recent years, immunotherapies aimed at reconstituting or enhancing the host immune response to control or even clear HBV have emerged as one of the most promising strategies for achieving functional cure.

**Methods:**

A systematic search of the Web of Science Core Collection (WoSCC) and Scopus databases identified 3,029 relevant articles published between January 2005 and December 2025. Using VOSviewer, CiteSpace, and Scimago Graphica as bibliometric tools, evaluation metrics were extracted or calculated to analyze and visualize the knowledge map. Publications were categorized by country, institution, author, journal, highly cited papers, and keywords. These variables were compared in terms of publication output and academic influence, including metrics such as citation counts, citation impact, H-index, and journal impact factor.

**Results:**

A total of 3,029 relevant publications were retrieved, originating from 116 countries or regions and 4188 research institutions. China and the United States led in both publication volume and impact; the most prolific institution was the Institut national de la santé et de la recherche médicale, followed by the University of London. Frontiers in Immunology was the most frequently cited journal; Janssen, Harry L A was the most prolific author, while Zoulim, Fabien had the highest H-index among all authors. Keyword clustering identified four primary categories: “functional cure”, “hepatocellular carcinoma”, “case report” and “advanced hepatocellular carcinoma”. Keyword and citation trends indicated three major research hotspots: persistent viral reservoirs, profound immune system depletion, and progression-driven mechanisms in hepatocellular carcinoma.

**Conclusions:**

This bibliometric analysis indicates that research on hepatitis B immunotherapy has experienced rapid development over the past two decades and is projected to grow rapidly toward the goal of “functional cure” in the coming years. Literature trends suggest that combination therapy strategies involving drugs with different mechanisms of action have been increasingly investigated for achieving functional cure for hepatitis B. Furthermore, comparative analysis of research trends across various immunomodulatory treatment regimens will contribute to a more comprehensive understanding of investigational therapeutic pathways in the near future.

## Introduction

Hepatitis B virus (HBV) infection represents an urgent global health challenge. According to World Health Organization estimates, nearly 296 million people worldwide are chronically infected, with HBV-related cirrhosis and hepatocellular carcinoma causing approximately 820,000 deaths annually (1, 2). During the natural course of chronic HBV infection, the virus establishes a stable reservoir of covalently closed circular DNA (cccDNA) within infected hepatocyte nuclei, enabling sustained viral antigen transcription. This mechanism represents one of the fundamental reasons for the difficulty in achieving complete viral clearance (3). Furthermore, prolonged exposure to high levels of viral antigens induces progressive functional exhaustion in the host immune system—particularly in virus-specific CD8^+^ T cells. This manifests as loss of effector function, upregulation of inhibitory receptors, metabolic abnormalities, and impaired memory cell formation, ultimately preventing effective clearance of infected cells (4, 5). This state of immune exhaustion constitutes the key immunological basis for persistent HBV infection and chronicity.

The current first-line treatment for chronic hepatitis B primarily relies on nucleos(t)ide analogues (NUCs), such as entecavir and tenofovir, which effectively suppress viral replication, improve liver histology, and partially restore T-cell function (6). However, NUCs fail to clear cccDNA within hepatocytes, leading to easy viral reactivation after discontinuation and extremely low HBsAg clearance rates—less than 10% after 5 years of treatment (7). Pegylated interferon-α (Peg-IFN) remains the only approved immunomodulatory agent for chronic hepatitis B, and it can achieve approximately 10–20% HBsAg clearance. However, its use is substantially limited by significant adverse reactions and poor tolerability (8). Accordingly, newer immunotherapies—such as immune checkpoint inhibitors and therapeutic vaccines—are being designed not as isolated alternatives, but as strategies intended to supplement or exceed the efficacy of interferon-based therapy, offering more targeted immune reconstitution with the potential for improved safety and tolerability. Consequently, there is an urgent clinical need for novel therapeutic strategies that can shorten NUC treatment duration and enhance HBsAg clearance rates.

Against this backdrop, immunotherapy aimed at restoring or enhancing the host’s specific immune response against HBV has emerged as one of the most promising strategies for achieving “functional cure” (9). Unlike traditional antiviral drugs, immunotherapy reverses immune tolerance and exhaustion through multiple mechanisms, primarily including: immune checkpoint inhibitors, such as anti-PD-1/PD-L1 antibodies, which restore T cell proliferation and effector function by blocking inhibitory signals; therapeutic vaccines, such as DNA vaccines or viral vector vaccines, which activate specific T cell and B cell responses by delivering HBV antigens; metabolic modulation therapies aimed at correcting mitochondrial dysfunction and oxidative stress in exhausted T cells; and genetically engineered adoptive T-cell therapies, such as T-cell receptor (TCR)- or chimeric antigen receptor (CAR)-modified T cells, which directly provide immune cells with potent antiviral activity (10, 11). It is essential to underscore that all these emerging immunotherapy strategies are predicated on a foundation of potent antiviral suppression, typically achieved with nucleos(t)ide analogues. This embodies the ‘suppress first, then modulate’ paradigm, ensuring that the host immune system is engaged only after viral replication is well controlled, which is critical for both safety and efficacy.

In recent years, with the rapid advancement of immunology, molecular biology, and gene editing technologies, research in the field of HBV immunotherapy has experienced explosive growth. This encompasses a complete innovation chain spanning fundamental mechanism exploration, novel target identification, drug design and optimization, early-stage clinical trials, and combination therapy evaluations (12–14). Despite this progress, there remains a lack of systematic overviews addressing the field’s knowledge structure, distribution of research capabilities, evolving research hotspots, and future trends from a macro perspective.

As a quantitative method for analyzing academic literature, bibliometrics effectively reveals disciplinary trends, collaborative networks, and research frontiers, providing holistic insights for field development (15). Therefore, this study aims to conduct a comprehensive bibliometric and visualization analysis of HBV immunotherapy-related research from 2005 to 2025. It seeks to systematically map the knowledge landscape of this field, identify core research forces, and trace the evolution of research hotspots. This analysis is intended to provide valuable reference for future basic research, clinical translation and policy formulation.

## Methods

### Data collection

This study was conducted as a systematic review integrated with bibliometric analysis following relevant reporting guidelines. The research question was defined to identify global research trends, hotspots, and frontiers in immunotherapy for chronic hepatitis B. A systematic literature search and two-step screening process were performed, with clear eligibility criteria focusing on population, concept, and context to ensure rigor and reproducibility.

WoSCC is renowned for its rigorous journal selection and reliable citation tracking, effectively capturing the impact and dissemination of scholarly work. Scopus excels in supporting interdisciplinary research through its extensive subject coverage and advanced citation tools. Combining WoSCC and Scopus provides a more comprehensive and accurate bibliometric analysis, offering deeper insights into research trends and academic developments.

**Figure 1:**
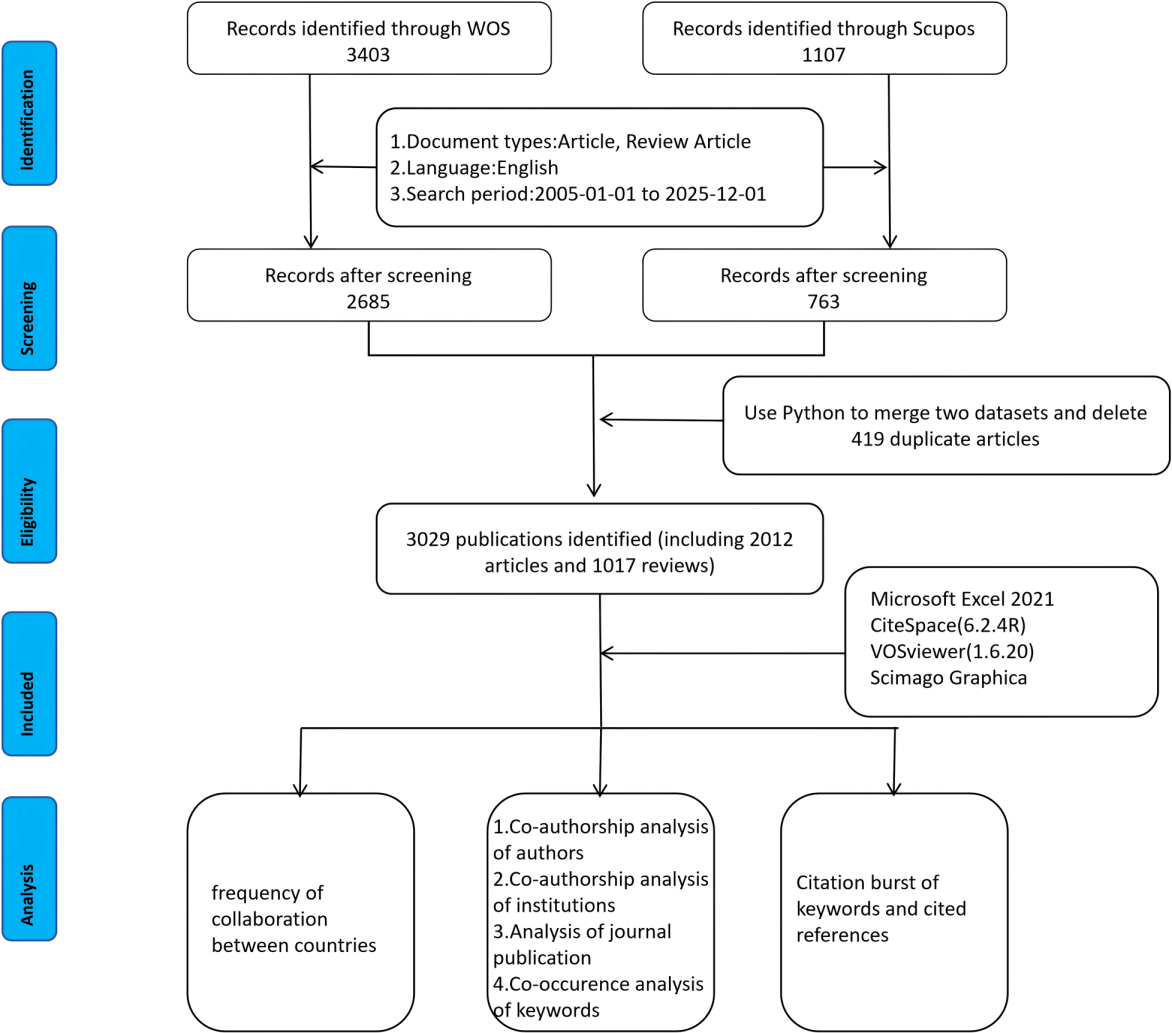
Flow-chart of the study.

**Figure 1** provides detailed specifications of data retrieval and exclusion criteria. Initially, to obtain bibliometric data, we selected the WoSCC and Scopus databases as our primary research databases. We retrieved relevant papers published between January 2005 and December 2025. Hepatitis B-related terms = (“chronic hepatitis B” OR “CHB disease” OR “CHB infection” OR “chronic HBV disease” OR “chronic HBV hepatitis” OR “chronic HBV infection” OR “chronic hepatitis B virus” OR “chronic infection of HBV” OR “chronic infection of hepatitis B” OR “chronic viral hepatitis B” OR “HBV chronic hepatitis” OR “HBV chronic infection”). Immune-related terms=(“immunotherapy” OR “immune therapy” OR “immunization therapy”). To facilitate further content analysis, only articles and review articles were included. Complete records and cited references were then extracted from relevant publications and saved in plain text format for subsequent analysis. Finally, 2,685 documents remained in WoSCC and 763 in Scopus.

Additionally, Python was used to merge the two datasets. Author names, institutions, and countries were standardized by correcting spelling variations and unifying naming formats. Duplicate records were identified by matching titles, authors, publication years, and DOIs; records without DOIs were verified using title and first author. All potential duplicates were manually checked to ensure accuracy. After this quality control step, a total of 3,029 unique studies were included for analysis.

### Study selection

We clarify that PRISMA guidelines are not fully applicable to this bibliometric-based study, since bibliometric analysis focuses on publication trends, research hotspots, and academic networks rather than clinical intervention effects, risk of bias, or patient-level evidence synthesis. Two reviewers independently screened titles, abstracts, and full texts based on predefined eligibility criteria. Discrepancies were resolved through discussion to reach consensus. Eligibility and exclusion criteria were clearly defined, and the full screening process is presented in the study flow chart.

### Bibliometric and scientometric analysis

This study utilized several bibliometric software tools for analysis and visualization: VOSviewer (version 1.6.20), CiteSpace (version 6.2.4R), Scimago Graphica, and Microsoft Office Excel 2021. Detailed parameters and thresholds were set as follows: For VOSviewer, minimum publication thresholds were ≥10 (countries), ≥20 (institutions), and ≥12 (authors); keyword co-occurrence was set at ≥150 occurrences, with modularity clustering (resolution = 1.0) and fractional counting for network normalization. For CiteSpace, time slices were set to 1 year (2005–2025), burst detection used a strength threshold ≥20 and minimum duration of 2 years, with the top 50% of nodes included; clustering employed the log-likelihood ratio (LLR) algorithm and pruning sliced networks for optimization. Scimago Graphica was used to map international collaboration with a minimum frequency threshold of 5. Full counting was used for publication counts and fractional counting for network analysis. VOSviewer was used to construct and visualize co-occurrence networks of countries, institutions, authors, and keywords. CiteSpace was used to detect keyword and citation bursts to identify research frontiers. Scimago Graphica was used to visualize international collaboration patterns. All data were processed and analyzed using Microsoft Office Excel 2021.

## Results

### Quantitative analysis of publication

**Figure 2:**
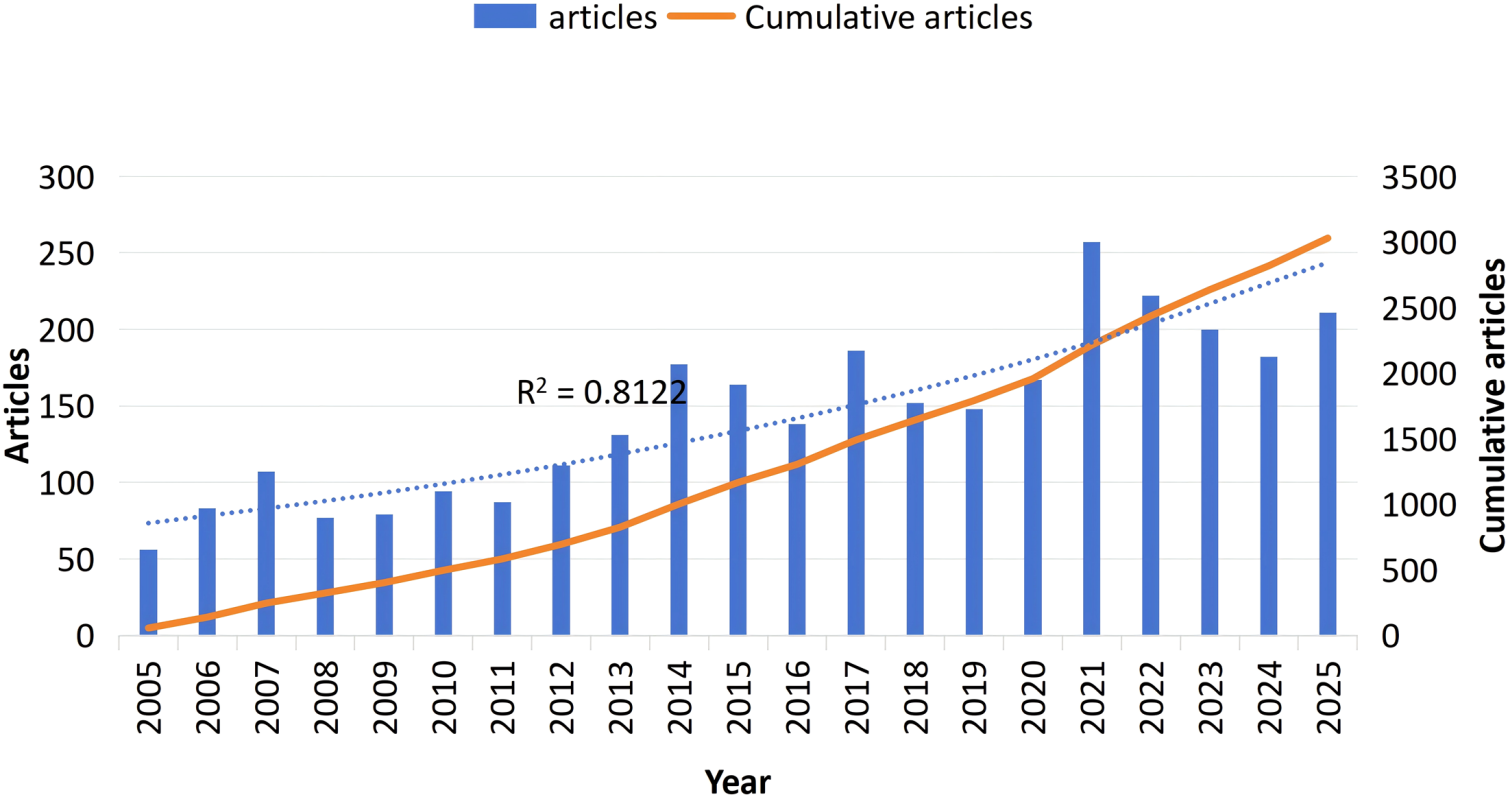
Number of publications per year and the cumulative number.

Based on the search terms, 3,029 articles were identified in WoSCC, including 2,012 original articles and 1,017 review articles. **Figure 2** shows the annual and cumulative publication counts related to hepatitis B. Publication numbers have steadily increased each year, from 56 articles in 2005 to 211 articles in 2025. Notably, over 200 related publications have been issued annually since 2020, reflecting heightened research interest in this topic. The year 2021 saw the highest publication volume with 257 articles.

### Analysis of national publication

**Figure 3:**
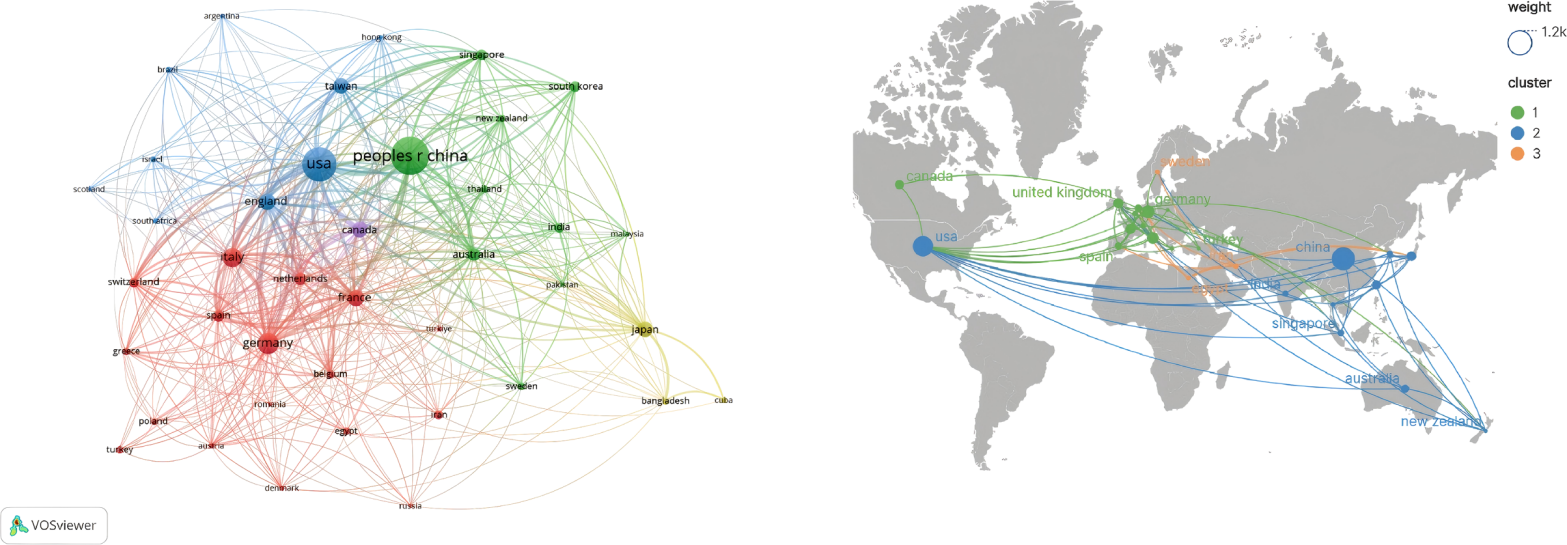
Network of cooperation in each country.

These publications originate from 116 countries or regions. **Table 1** lists the top 10 countries by publication volume, with six of them located in Europe and the Americas. Publications from the top three countries account for over 60% of the total (n=1982, 65.4%), including China in Asia (n=952, 31.5%), the United States in North America (n=736, 24.3%), and Germany in Europe (n=294, 9.7%). Using VOSviewer to filter and visualize countries with 10 or more publications, **Figure 3** presents each country’s collaborative network based on publication volume and relationships.

**Table 1:** Top 10 countries for publications.

Rank	Country	Article	Citations	Total link strength	H-index
1	People's Republic of China	952	29768	443	69
2	USA	736	48481	728	95
3	Germany	294	19318	441	62
4	Italy	241	12370	308	49
5	France	180	13216	315	47
6	England	176	13179	391	48
7	Japan	159	9619	161	34
8	Canada	155	6984	257	38
9	Taiwan	153	13374	168	44
10	Australia	116	8815	223	39

### Analysis of institution publications

**Figure 4:**
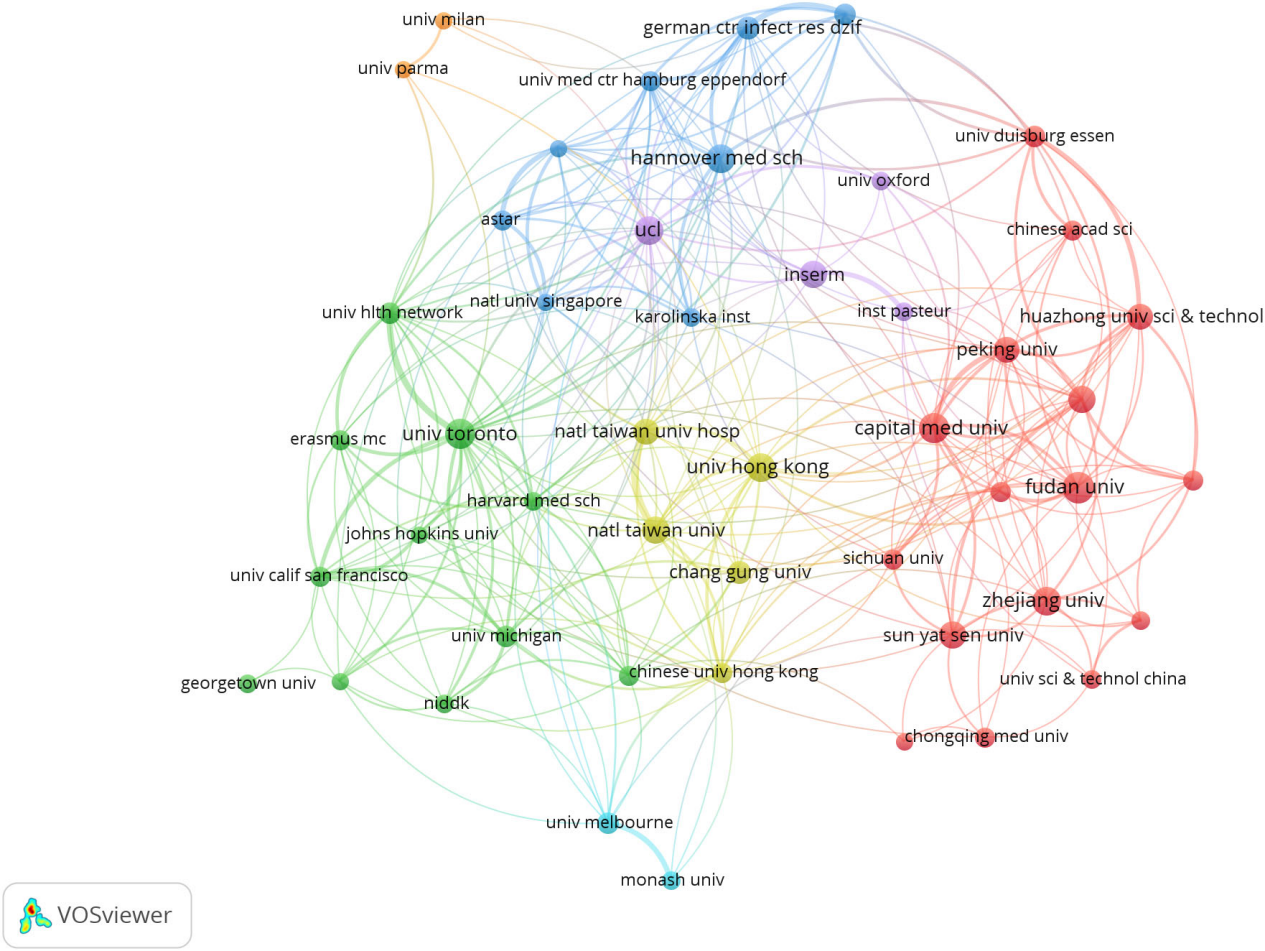
Key institutions and their relationships in this area.

Research on immunotherapy for chronic hepatitis B is conducted at approximately 4188 institutions worldwide. **Table 2** lists the top 10 institutions by publication output. The Institut national de la santé et de la recherche médicale in France published the highest number of papers at 128, followed by the University of London in the United Kingdom with 89 publications. **Figure 4** displays major institutions with over 20 publications and their collaborative relationships within this field.

**Table 2:** Top 10 institutions for literature output.

Rank	Organization	Country	Article	H-index	Citations
1	Institut national de la santé et de la recherche médicale	France	128	43	10093
2	University of London	England	89	42	8944
3	University of Toronto	Canada	78	29	2680
4	Fudan University	People's Republic of China	72	20	1858
5	National Taiwan University	Taiwan	71	34	8638
6	USA National Institutes of Health	USA	70	37	6088
7	Capital Medical University	People's Republic of China	69	18	1412
8	Erasmus University Rotterdam	Netherlands	68	26	1916
9	Erasmus MC	Netherlands	66	25	1827
10	University College London	England	66	37	3914

### Analysis of journals

A total of 802 journals have published articles on research in the field of immunotherapy for chronic hepatitis B. **Table 3** displays the top 10 journals ranked by publication volume, with Frontiers in Immunology leading at 115 articles and an H-index of 27. Among the top 10 journals by H-index, Journal of Hepatology holds the highest H-index at 55, ranking second with 107 articles and an impact factor of 33.0.

**Table 3:** Top 10 journals ranked by publication quantity.

Rank	Journal	Article	2024IF	JCR-c	Total citations	H-index
1	Frontiers in Immunology	115	5.9	Q1	3092	27
2	Journal of Hepatology	107	33.0	Q1	11264	55
3	World Journal Of Gastroenterology	93	5.4	Q1	3070	31
4	Hepatology	71	15.8	Q1	8514	37
5	Journal of Viral Hepatitis	70	2.3	Q3	1613	21
6	Liver International	64	5.2	Q1	2113	25
7	Antiviral Research	57	4.0	Q1	1761	23
8	Plos One	49	2.6	Q2	1413	20
9	Viruses-Basel	36	3.4	Q1	755	15
10	Journal of Virology	35	3.8	Q2	2181	21

### Analysis of author influence and collaboration

**Figure 5:**
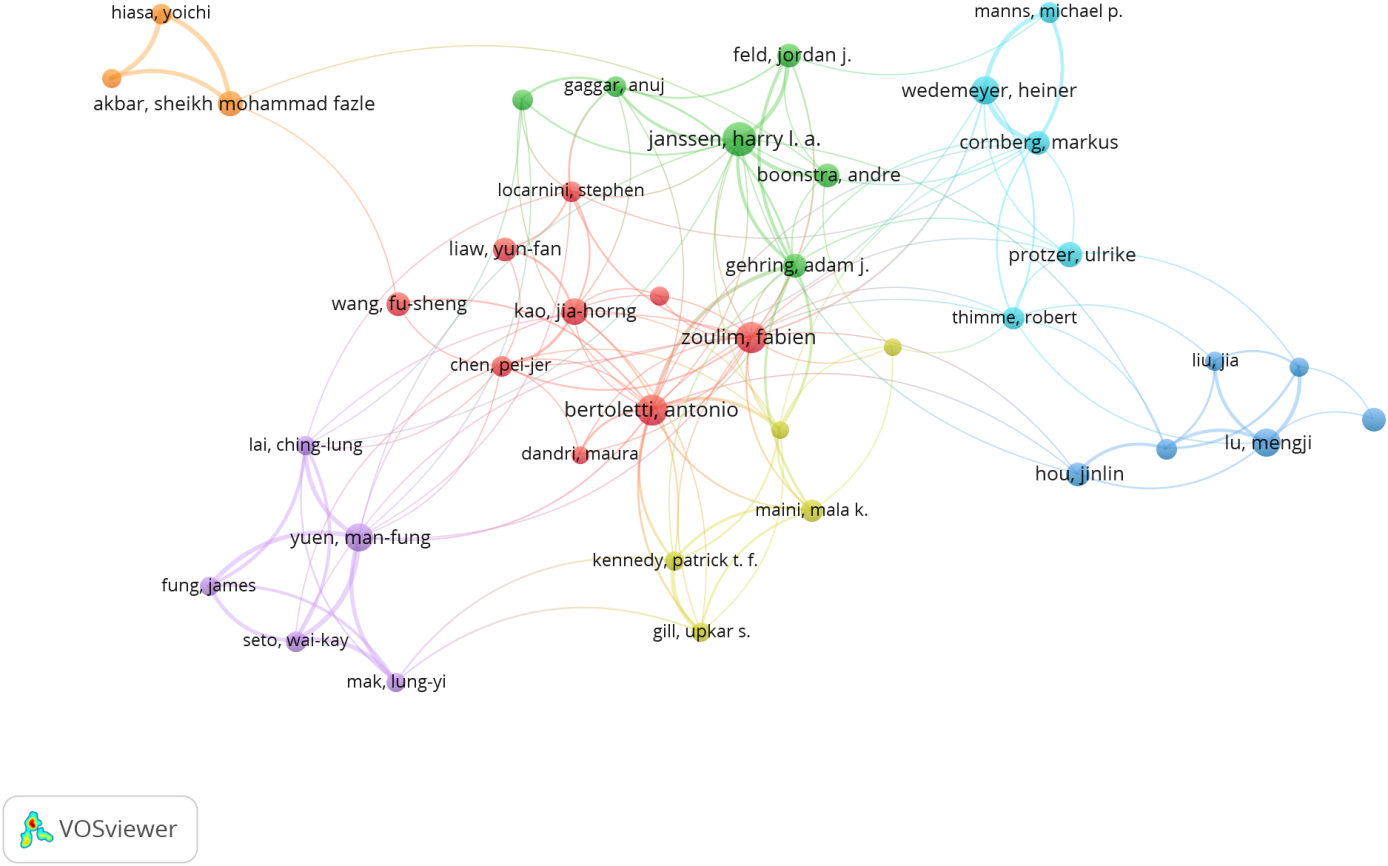
Collaborative relationships among researchers.

A total of 14,490 authors contributed to research on immunotherapy for chronic hepatitis B. **Table 4** lists the top 10 authors by publication volume, all of whom published at least 20 papers. Harry L. A. Janssen ranked first with 47 publications, an h-index of 89, and 2,362 total citations. followed by Bertoletti, Antonio, with 37 publications, an h-index of 73, and 3,327 total citations. We constructed a collaboration network based on 41 authors with at least 12 publications. **Figure 5** illustrates researchers’ collaborative relationships, where node size represents publication volume, color indicates publication era, and line thickness reflects collaboration frequency. This reveals that leading authors have formed seven primary collaborative clusters.

**Table 4:** The top 10 authors in the number of publications.

Rank	Author	Country	Article	H-index	Total citations	Total link strength
1	Janssen, Harry L A	Netherlands	47	89	2362	109
2	Bertoletti, Antonio	Singapore	37	73	3327	112
3	Zoulim, Fabien	France	35	97	3036	88
4	Wedemeyer, Heiner	Germany	32	92	1610	73
5	Lu, Mengji	People's Republic of China	29	50	1417	117
6	Yuen, Man-Fung	People's Republic of China	28	91	1512	92
7	Protzer, Ulrike	Germany	26	58	926	25
8	Cornberg, Markus	Germany	22	52	1509	59
9	Boonstra, Andre	Netherlands	21	43	617	32
10	Kao, Jia-Horng	Taiwan	21	81	2841	66

### Reference bursts detection

**Figure 6:**
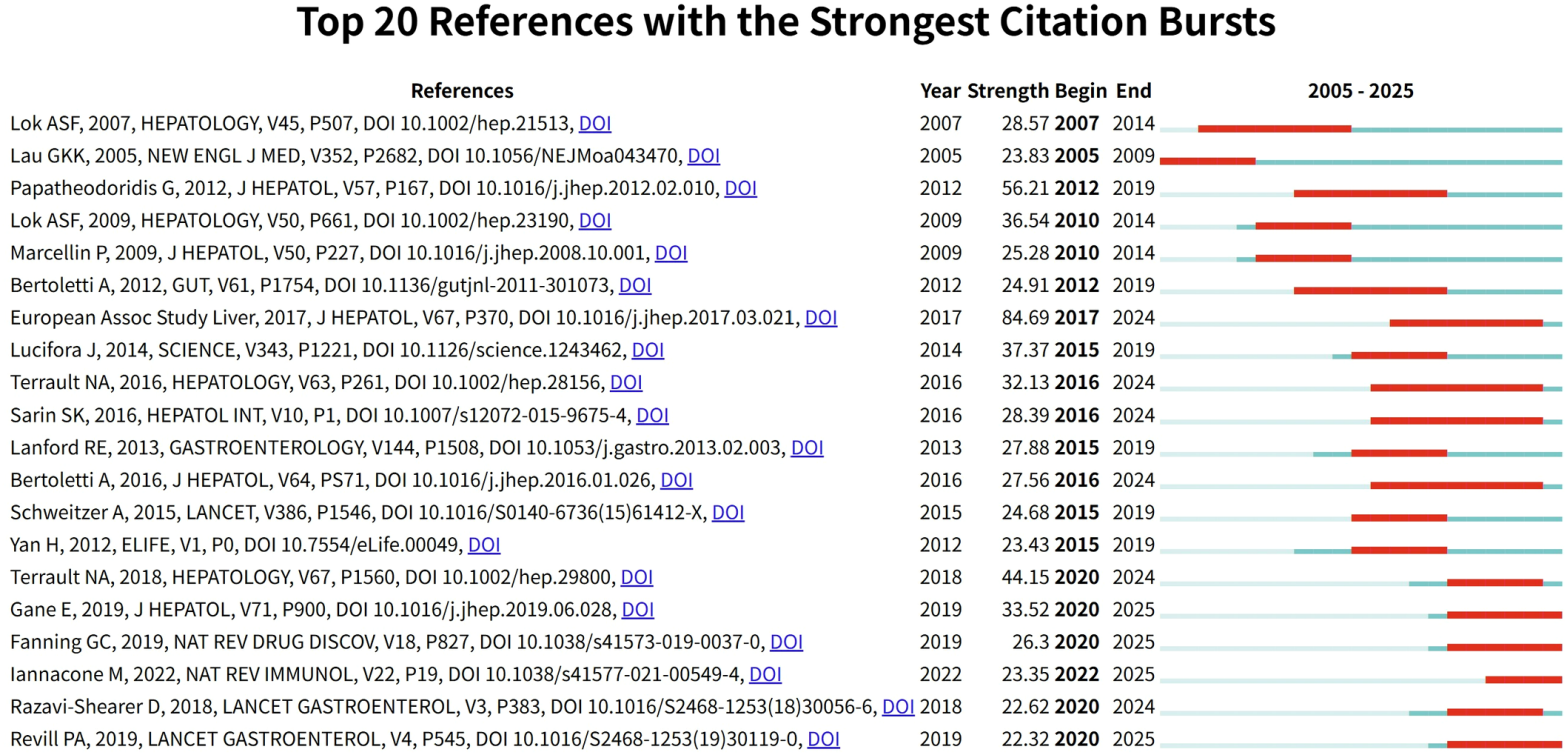
Top 20 references with the strongest citation bursts.

A citation explosion refers to a sharp increase in citation frequency following a paper’s publication, indicating heightened interest in the related topic. **Figure 6** displays the top 20 references with the strongest citation explosions, where the dark blue line represents citation frequency from 2005 to 2025, and the red line indicates the range of the citation explosion. The article “EASL 2017 Clinical Practice Guidelines on the Management of Hepatitis B Virus Infection” (16), published in the Journal of Hepatology, exhibited the highest citation burst value from 2005 to 2025, with strength = 84.69 and burst period = 2017–2024. The second strongest citation burst was for the paper titled “EASL clinical practice guidelines: Management of chronic hepatitis B virus infection” (17), also published in the Journal of Hepatology (strength = 56.21, burst period = 2012–2019). Among the top 20 papers, 4 are still experiencing ongoing citation bursts. Research primarily focused on HBV infection immune mechanisms, immunotherapy strategies such as PD-1 inhibitors, and global exploration of cure pathways.

### Keyword analysis and analysis of keywords bursts

**Figure 7:**
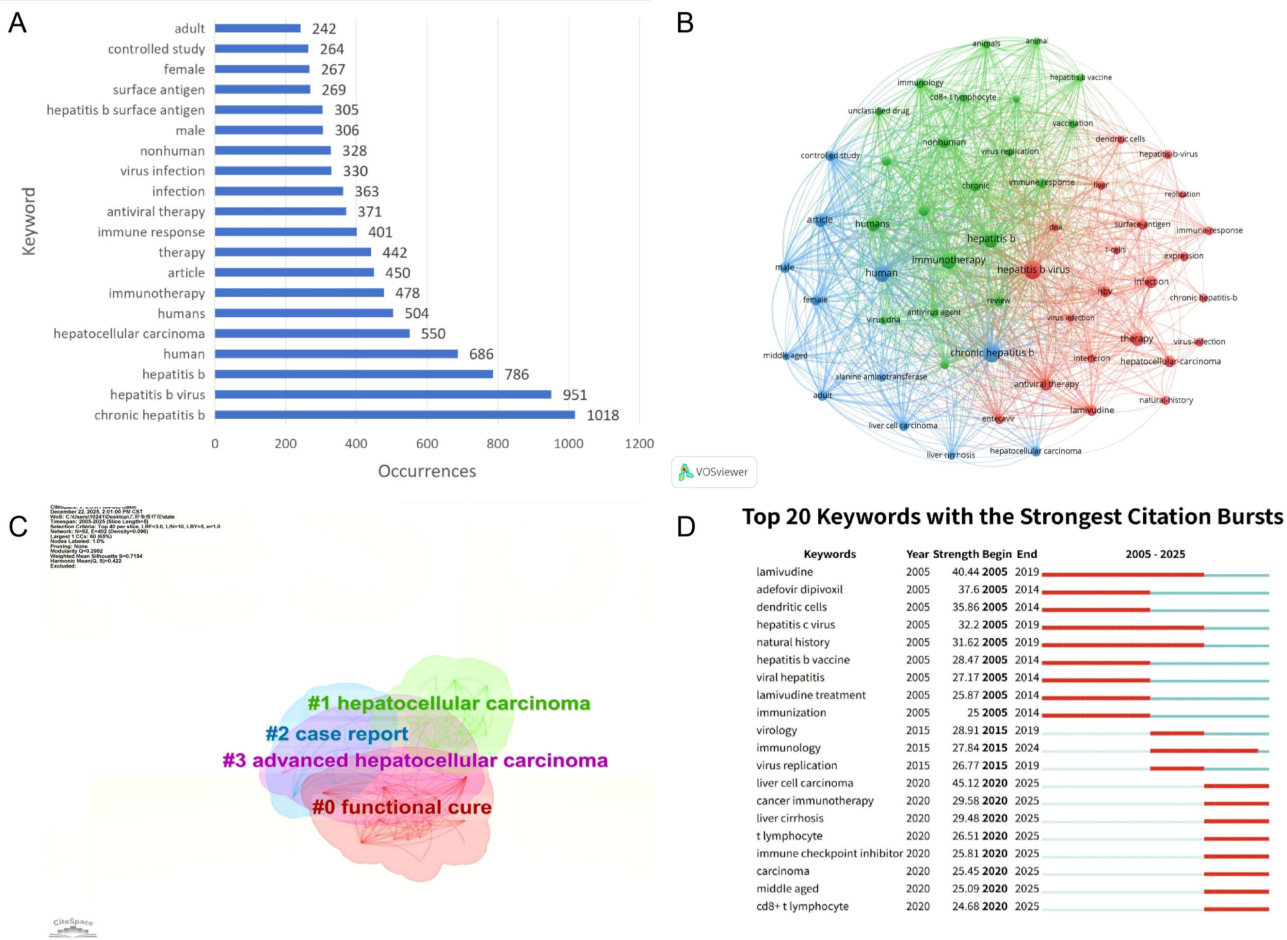
**(A)** A list of the 20 most frequently used keywords. **(B)** Keyword co-occurrence network. **(C)** Cluster analysis of keywords. **(D)** The top 20 keywords with the strongest citation burst.

Keyword co-occurrence analysis helps identify research hotspots in chronic hepatitis B immunotherapy. **Figure 7A** displays the top 20 keywords ranked by frequency. The most frequently occurring keyword is “chronic hepatitis B” appearing 1,018 times. Among the 3,029 papers, 55 keywords with frequencies ≥150 were extracted and clustered. **Figure 7B** presents a network visualization of these keywords. Node size reflects keyword frequency, with terms grouped into three distinct clusters. **Figure 7C** shows the results of keyword cluster analysis, with each cluster clearly labeled to reflect its core research theme. The clustering results indicate that research in this domain is concentrated on three major directions: functional cure of chronic hepatitis B, immune reconstitution and T-cell exhaustion, and hepatocellular carcinoma progression and related immunotherapy. These clusters are highly consistent with the key research hotspots and conclusions of this study, confirming that the core focus of global research has shifted toward achieving functional cure through immune modulation and addressing immune exhaustion in HBV infection. **Figure 7D** lists the top 20 keywords with the strongest bursts. The keyword “liver cell carcinoma” received the highest attention, with strength = 45.12 and burst period = 2020–2025. It was followed by “lamivudine” with strength = 40.44 and burst period = 2005–2019. Recently, keywords such as “cancer immunotherapy” (2020–2025), “T lymphocyte” (2020–2025), “immune checkpoint inhibitor” (2020–2025) and “CD8+ T lymphocyte” (2020–2025) remain in active use, indicating that future research focus will continue to center on these topics.

## Discussion

### Principal findings

This study employed bibliometric methods to systematically analyze research trends in the field of immunotherapy for chronic hepatitis B from 2005 to 2025. Results indicate sustained rapid growth in research output, with annual publications increasing from 56 in 2005 to 211 in 2025. A period of accelerated growth commenced after 2020, stabilizing above 200 publications annually. This trend is driven by multiple factors: Persistent global burden of chronic HBV infection, where functional cure remains the fundamental clinical goal, is now met by rapid advances in immunology and virology research, which have laid the theoretical foundation for developing novel immunotherapies such as therapeutic vaccines and immune checkpoint inhibitors; and the introduction of combination therapy strategies alongside progressive clinical trials have further propelled a paradigm shift in the field—from solely suppressing viral replication toward systemic immune reconstitution.

An analysis of 3,029 global publications reveals that research on immunotherapy for chronic hepatitis B exhibits a high degree of internationalization and collaboration, with China and the United States forming the core of the cooperative network in this field. Current research consensus emphasizes that achieving functional cure for hepatitis B relies not only on effectively suppressing viral replication but also on rebuilding or enhancing host-specific immune responses to reverse immune tolerance and exhaustion states (18). Accordingly, the guiding principle of ‘suppress first, then modulate’ has gained wide acceptance: deep and sustained viral suppression with nucleos(t)ide analogues is regarded as a prerequisite before initiating immunotherapy, in order to minimize the risk of severe immune-mediated hepatitis flares and to provide a controlled immunological environment in which immune reconstitution can safely occur. Research frontiers are concentrated on two core strategies—therapeutic vaccines and immune checkpoint inhibitors—with active exploration of their combination with existing antiviral drugs to synergistically activate T-cell and B-cell immunity, thereby achieving sustained immune control (19). It is important to recognize that these cutting-edge approaches are positioned to extend, rather than simply replace, the immunomodulatory capabilities of pegylated interferon-α. While interferon has demonstrated the proof-of-concept that immune activation can lead to functional cure, its limitations have fueled the search for agents that can more precisely and safely reverse HBV-specific immune exhaustion. Therefore, the next generation of immunotherapies is rationally designed to either synergize with Peg-IFN in combination regimens or to ultimately surpass its effectiveness as standalone immune-based interventions.

Analysis of countries and regions reveals the distribution patterns and collaboration trends of global scientific research capabilities. China and the United States lead in both total publications and total citations, reflecting their resource investments and academic influence in liver disease immunology research. However, international collaboration networks indicate tighter cooperation among European and American nations, forming research clusters centered around France, Germany, and the United States. This suggests that while maintaining high productivity, Asian countries should further strengthen in-depth collaborations with top European and American teams to enhance research’s international impact and translation efficiency.

Institutional collaboration network analysis reveals that chronic hepatitis B immunotherapy research has formed several cross-regional research clusters centered around internationally leading institutions. European institutions such as Inserm, Karolinska Institutet, and the University of Oxford occupy pivotal positions within the network, maintaining close collaborations with numerous European and global institutions. Concurrently, Asian research forces hold significant standing within this network. Institutions such as Peking University, Fudan University, Huazhong University of Science and Technology in China, alongside the University of Hong Kong and the National University of Singapore, not only demonstrate active research output but have also established academic connections with central nodes in Europe and the United States through international collaboration, forming transcontinental cooperation pathways. Overall, this collaborative network exhibits structural characteristics of multi-centrism, cross-regionality, and synergistic interaction, reflecting the ongoing trend toward international integration of research resources and knowledge sharing in this field.

Author collaboration network analysis further confirms that multiple tightly interconnected academic communities have emerged within this field, with high-output authors such as Janssen, Harry L A and Zoulim, Fabien often serving as core nodes within these communities. Notably, despite China’s lead in total publication volume, it remains relatively weak in terms of top high-output authors and international collaboration networks. This suggests that Chinese research still has room for improvement in cultivating globally influential academic leaders and developing original theoretical frameworks.

Regarding journal impact, Q1-ranked journals dominate the top ten by publication volume, indicating a consistent convergence of high-quality research toward high-impact platforms. Frontiers in Immunology ranks highest in publication volume, while Journal of Hepatology holds the highest H-index, reflecting the central role of immunology and hepatology journals in disseminating knowledge within the field.

Analysis of highly cited and rapidly emerging publications not only identifies landmark studies in the field but also reveals key pathways in the evolution of understanding. The most rapidly cited literature centers on the review and consensus statement “EASL 2017 Clinical Practice Guidelines on the management of hepatitis B virus infection” (16), published in the Journal of Hepatology, marking the formal shift in clinical treatment goals from “viral suppression” to “functional cure” Key terms that have surged in recent years—such as “immune checkpoint inhibitor”, “functional cure” and “CD8+ T lymphocyte”—clearly point to the central role of immunotherapy in achieving cure goals. The evolution of these keywords further reflects a shift in research focus: early studies centered on traditional therapies like “lamivudine” and “pegylated interferon.”, while recent research has concentrated on immunotherapy-related themes such as “cancer immunotherapy”,”T lymphocyte” and “immune checkpoint inhibitor”. This indicates a transition from purely antiviral strategies toward exploring cure pathways centered on immune reconstitution.

### The immune mechanism of hepatitis B cure: from exhaustion to reconstruction

The “functional cure” of chronic hepatitis B, defined as sustained clearance of hepatitis B surface antigen (HBsAg), relies not only on effective viral suppression but more critically on rebuilding the host’s specific immune response against HBV. In recent years, immunotherapy has emerged as a core strategy for achieving this goal, particularly through immune checkpoint inhibitors and therapeutic vaccines. Their mechanisms of action primarily revolve around reversing T-cell exhaustion, rebuilding antigen-specific immune memory, and breaking immune tolerance states (20).

During chronic HBV infection, virus-specific CD8^+^ T cells are continuously exposed to high antigen loads, gradually entering an “exhausted” state characterized by loss of effector function, reduced proliferative capacity, and high expression of inhibitory receptors such as PD-1, CTLA-4, and TIM-3. Among these, the interaction between PD-1 and its ligand PD-L1 is one of the key pathways mediating T cell functional suppression (21). Blocking the PD-1/PD-L1 axis restores T cell proliferation, cytokine secretion, and cytotoxic activity, thereby reactivating immune clearance of infected hepatocytes. A Phase Ib study using nivolumab demonstrated that even in HBeAg-negative patients with suppressed viral load, a single low-dose nivolumab infusion induced HBsAg decline in most subjects. including one case achieving HBsAg clearance with seroconversion to HBsAb, accompanied by transient ALT elevation and peripheral HBV-specific T cell response reconstitution, suggesting immune-mediated hepatic inflammation and immune system reactivation (22).

Therapeutic vaccines aim to break immune tolerance by delivering HBV antigens to activate naive or memory T cells and B cells (23, 24). For example, GS-4774 is a yeast-expressed HBV polyantigen fusion vaccine designed to elicit multispecific T cell responses (25). Although it demonstrated immunogenicity in early trials, subsequent studies failed to show significant reduction in HBsAg levels, suggesting limitations in its standalone application for reversing immune exhaustion.

The combination strategy of “vaccine + checkpoint inhibitor” is considered a more promising curative pathway: vaccines provide antigenic stimulation to expand the pool of specific T cells, while checkpoint inhibitors relieve immune suppression to enhance T cell function (12). Although no significant synergistic effect was observed in the study of nivolumab combined with GS-4774, this may be related to vaccine design, administration timing, or patient immune status. Future efforts should focus on optimizing combination regimens, such as administering vaccines after checkpoint blockade or combining multiple immunomodulators to synergistically reverse immune exhaustion and reestablish sustained antiviral immune responses.

Current immunotherapy still faces challenges including safety concerns, response heterogeneity, and difficulties in monitoring hepatic immunity. Future research should focus on developing personalized treatment strategies based on patient immune profiling, exploring novel immune targets such as TIM-3, LAG-3, and OX40, and advancing the translation of immunotherapy from basic research to clinical precision medicine through multi-omics technologies and analysis of the hepatic immune microenvironment (26).

### Comparisons with previous studies

Compared to previous research articles, this study clearly identifies a fundamental paradigm shift in research approaches—a systematic transition from traditional viral suppression strategies toward immune reconstitution aimed at achieving “functional cure” as the ultimate goal. This transformative shift is vividly reflected in the recent explosive growth of keywords such as “functional cure” and “immune checkpoint inhibitors.” Second, the analysis highlights the significant expansion in both depth and breadth of interdisciplinary collaboration networks. The convergence of immunology, virology, bioengineering, and clinical hepatology has become the core engine driving therapeutic innovation. Crucially, this study precisely captures how current research focus is tightly centered on two core immunological intervention strategies—therapeutic vaccines and immune checkpoint inhibitors—along with their synergistic combination regimens. These approaches aim to reverse immune exhaustion and rebuild sustained, effective antiviral immune responses. These findings not only deepen our understanding of the evolving knowledge landscape in chronic hepatitis B immunotherapy but also establish clear pathways and priorities for future academic exploration and clinical translation research. Specifically, they point toward optimizing combination immunotherapy regimens to ultimately achieve functional cure in chronic hepatitis B.

### Strengths and limitations

This study offers the inaugural systematic visualization of hepatitis B immunotherapy research through multi-software bibliometric analysis, supplying trend and hotspot insights to guide future investigations. Secondly, it allows researchers to promptly pinpoint key countries, institutions, authors, and influential publications, enabling an efficient grasp of emerging directions. Thirdly, by employing keyword co-occurrence and burst detection analyses, the work delineates the domain’s progression from foundational studies to clinical practice and from single therapies to combined regimens. Lastly, it furnishes scientific administrators and clinicians with a synthesized perspective on the field, supporting optimized resource distribution and clearer research prioritization.

Several limitations of this review are recognized. First, as a bibliometric inquiry, the work depends substantially on software for data collection and processing; while such analysis cannot substitute for systematic reviews, it is effective in revealing macro-trends across large corpora. Second, data were drawn exclusively from the Web of Science Core Collection and Scopus, which is a typical constraint in bibliometric studies. This means that pertinent studies indexed in other databases, such as PubMed or EMBASE, may have been omitted, introducing potential selection bias. Third, the rise in publications on chronic hepatitis B immunotherapy from 2005 to 2025 may be attributable in part to the broader expansion of scientific output in biomedicine during this period. Finally, due to citation delays, recently published high-impact breakthrough studies may not be fully captured in the current analytical metrics.

### Clinical implications for clinicians and policy-makers

For clinicians, current research trends clearly point toward immune reconstitution targeting “functional cure”. Clinicians should monitor clinical trial progress of immune checkpoint inhibitors and therapeutic vaccines, particularly their combination regimens with existing antiviral medications. In clinical practice, physicians can progressively implement personalized immunotherapy assessments based on patients’ immune status, virological indicators, and liver histological features. Additionally, identifying populations likely to benefit from immunotherapy—such as HBeAg-negative patients—and closely monitoring and managing immune-related adverse events during treatment are critical for achieving safe and effective outcomes.

Policy makers should recognize the strategic importance of immunotherapy within the chronic hepatitis B treatment framework and increase funding for related basic and clinical research, particularly for advancing multinational, multicenter clinical trials. This study indicates that China and the United States lead in research, yet international collaboration networks remain centered in Europe and North America. Asian countries are advised to strengthen cooperation with top international teams to promote knowledge sharing and resource integration. Concurrently, establishing standardized immunotherapy evaluation systems and long-term follow-up databases should be encouraged to provide real-world evidence supporting guideline updates and healthcare policy development.

In summary, the findings of this study not only assist clinicians in grasping treatment frontiers and optimizing therapeutic decisions but also provide policymakers with a basis for resource allocation, research planning, and international collaboration. Ultimately, this will propel chronic hepatitis B treatment toward higher-level cure goals and enhance overall public health prevention and control capabilities.

## Conclusion

In summary, research on immunotherapy for hepatitis B infection has garnered increasing attention, with a steady rise in published literature over the past two decades underscoring the field’s growing significance. This study employs bibliometric and visualization techniques to map the developmental trajectory and structural shifts within this domain over the last twenty years. Literature trends suggest that future research will continue to focus on the goal of “functional cure” for hepatitis B, with investigational strategies centered on combination therapies incorporating novel immunotherapies such as immune checkpoint inhibitors. This bibliometric analysis enables scientists to gain a clearer understanding of the knowledge framework and collaborative networks within the field, laying the groundwork for deeper mechanistic exploration and clinical translation. It is hoped that subsequent research findings will enhance clinical management of chronic hepatitis B patients, ultimately supporting progress toward functional cure and improved patient outcomes.

## Data Availability

The original contributions presented in the study are included in the article/supplementary material. Further inquiries can be directed to the corresponding authors.
